# C_60_ Fullerenes Suppress Reactive Oxygen Species Toxicity Damage in Boar Sperm

**DOI:** 10.1007/s40820-019-0334-5

**Published:** 2019-11-27

**Authors:** Xinhong Li, Lirui Wang, Huan Liu, Jieli Fu, Linqing Zhen, Yuhua Li, Yaozhong Zhang, Yafei Zhang

**Affiliations:** 1 Shanghai Key Laboratory of Veterinary Biotechnology, School of Agriculture and Biology, Shanghai Jiao Tong University, Shanghai, 200240 People’s Republic of China; 2 Institute of Nano Biomedicine and Engineering, Shanghai Engineering Research Centre for Intelligent Diagnosis and Treatment Instrument, Department of Instrument Science and Engineering, School of Electronic Information and Electrical Engineering, Shanghai Jiao Tong University, Shanghai, 200240 People’s Republic of China; 3 Department of Electrical and Computer Engineering, Michigan State University, East Lansing, USA; 4 Key Laboratory of Thin Film and Microfabrication (Ministry of Education), Department of Micro/Nano Electronics, School of Electronics, Information and Electrical Engineering, Shanghai Jiao Tong University, Shanghai, 200240 People’s Republic of China

**Keywords:** Carboxylated C_60_, Semen preservation, Oxidative stress, Motility, Protein dephosphorylation

## Abstract

We report the carboxylated C_60_ improved the survival and quality of boar sperm during liquid storage at 4 °C and thus propose the use of carboxylated C_60_ as a novel antioxidant semen extender supplement. Our results demonstrated that the sperm treated with 2 μg mL^−1^ carboxylated C_60_ had higher motility than the control group (58.6% and 35.4%, respectively; *P *˂ 0.05). Moreover, after incubation with carboxylated C_60_ for 10 days, acrosome integrity and mitochondrial activity of sperm increased by 18.1% and 34%, respectively, compared with that in the control group. Similarly, the antioxidation abilities and adenosine triphosphate levels in boar sperm treated with carboxylated C_60_ significantly increased (*P *˂ 0.05) compared with those in the control group. The presence of carboxylated C_60_ in semen extender increases sperm motility probably by suppressing reactive oxygen species (ROS) toxicity damage. Interestingly, carboxylated C_60_ could protect boar sperm from oxidative stress and energy deficiency by inhibiting the ROS-induced protein dephosphorylation via the cAMP-PKA signaling pathway. In addition, the safety of carboxylated C_60_ as an alternative antioxidant was also comprehensively evaluated by assessing the mean litter size and number of live offspring in the carboxylated C_60_ treatment group. Our findings confirm carboxylated C_60_ as a novel antioxidant agent and suggest its use as a semen extender supplement for assisted reproductive technology in domestic animals.

**Electronic supplementary material:**

The online version of this article (10.1007/s40820-019-0334-5) contains supplementary material, which is available to authorized users.

## Highlights

- Carboxylated C60 may be considered a novel antioxidant agent to be used as a semen extender supplement for assisted reproductive technology.
- Carboxylated C60 could enhance the motility parameters and characteristics of boar spermatozoa during liquid storage at 4 °C and protect boar sperm from oxidative stress by inhibiting the reactive-oxygen-species–induced protein dephosphorylation.
- Novel insights into the molecular mechanisms contributing to the protective effects of carboxylated C60 are provided.

**Electronic supplementary material:**

The online version of this article (10.1007/s40820-019-0334-5) contains supplementary material, which is available to authorized users.

## Graphical abstract

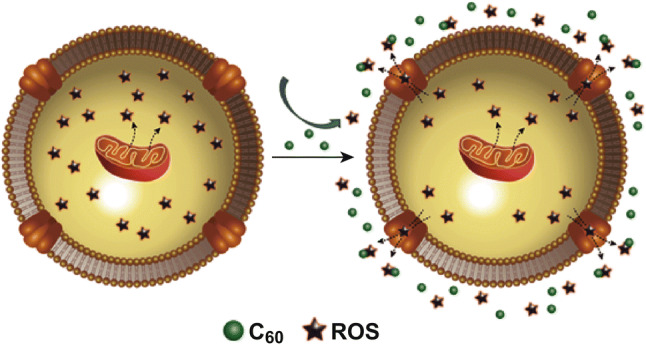

## Introduction

Fullerenes are typical zero-dimensional carbon nanomaterials and have been widely used for various purposes, including electrocatalysts [1, 2], energy storage [3, 4], photodetectors [5, 6], and solar cells [7–9]. Recently, carboxylated C_60_ has been shown to be one of the most important carbon nanoparticle derivatives and extensively studied in biomedical applications [10–12]. Notably, carboxylated C_60_ is known to be a putative antioxidant [13, 14] with a powerful bio-antioxidant ability, which can prevent tissue dysfunction induced by oxidative stress [15–17]. Previous studies have demonstrated that in various in vivo and in vitro pathological models the multidirectional positive biological effects of carboxylated C_60_ are mediated by its antiradical activity [18, 19]. Additionally, many beneficial effects of carboxylated C_60_ have been observed even at extremely low doses. Altogether, these data suggest that carboxylated C_60_ is a promising tool for the control of reactive oxygen species (ROS)-dependent pathological damage, including brain and immune system diseases. Despite substantial experimental results highlighting the positive effect of carboxylated C_60_ on biological systems in vivo and in vitro, few attempts have been made to clarify the molecular mechanism of its antioxidant action. In particular, the effects of carboxylated C_60_ on mammalian sperm motility and oxidative stress state remain unexplored.

Although carboxylated C_60_ is a potential non-enzymatic antioxidant, its practical production is still limited, especially in the biological field. In the modern pig husbandry, artificial insemination (AI) is extensively used worldwide [20]. Numerous studies have shown that the semen used for AI must be diluted with suitable extenders and should be preserved at 17 °C or in liquid nitrogen. However, the major drawback is the limited lifespan of the sperm, which can only be preserved for 5 days in vitro [21]. Additionally, although cryopreservation can prolong the semen storage time in vitro, only 1% of the artificial inseminations with frozen-thawed boar sperm is successful worldwide [22]. Thus, to avoid boar sperm from being subjected to freezing injury due to storage in liquid nitrogen and to prolong sperm survival time in vitro, many researchers are committed to studying liquid storage at 4–5 °C.

Due to a low cholesterol/phospholipid ratio, boar spermatozoa are particularly vulnerable to low temperatures, which can lead to oxidative stress due to the inappropriate formation of ROS [23]. It has previously been shown that overproduction of ROS has an adverse impact on sperm motility, viability, and morphology [24]. Therefore, regulation of the generation of excessive ROS to eliminate the accompanying ROS-mediated damage to the structural integrity of the sperm plasma and acrosome, and maintenance of the stability of the genetic material during external storage of boar semen [25], are serious challenges that urgently require solutions. In addition, because of the limited ability of sperm to establish a powerful defense system of antioxidants, substantial efforts have been invested to search for exogenous antioxidants to prevent oxidative damage [26, 27]. To date, studies have suggested that the addition of antioxidant agents to semen can protect sperm from ROS attack and improve sperm quality parameters [24, 28, 29]. However, the feasibility of the application of carboxylated C_60_ in the reproductive field of animal production is completely unknown, and in particular, little is known about the protective mechanisms of antioxidants with respect to ROS toxicity in sperm preserved at low temperatures. Moreover, the effect of exposure of sperm to carboxylated C_60_ on AI procedures has not been investigated. Therefore, it is extremely crucial to investigate whether boar sperm exposed to antioxidant materials prepared using carboxylated C_60_ can be used in current AI procedures.

The present study aimed to explore the effect of carboxylated C_60_ on spermatozoa, with a particular focus on the sperm functionality, and whether carboxylated C_60_ could exert significant antioxidant effects in boar spermatozoa during in vitro preservation at 4 °C. Herein, we performed a systematic assessment of the protective role of carboxylated C_60_ against ROS oxidative damage in boar sperm preserved at 4 °C. The tested viability and functional parameters included boar sperm motility, membrane and acrosome integrity, mitochondrial membrane potential, cAMP and ATP levels, ROS production, antioxidation indexes, and the protein phosphorylation level. Additionally, the safety of carboxylated C_60_ as an alternative antioxidant was also evaluated. Our results suggested that carboxylated C_60_ exerted no obvious toxicity toward boar sperm cells. Given these data, the good biocompatibility and antioxidative capacity of carboxylated C_60_ demonstrated its great potential used as a semen extender. Simultaneously, this study provides a theoretical basis for broadening the use of carbon nanomaterials as antioxidants in the field of livestock and animal breeding.

## Experimental Section

### Preparation of Hydrated C_60_ Fullerene

C_60_ samples with a purity of > 99.98% were obtained from Shanghai Jiao Tong University. The carboxylated C_60_ was prepared using the method described by Andrievsky, which is based on the transfer of fullerene from an organic solution into the aqueous phase by ultrasonic treatment, as described elsewhere [16]. The averaged FTLA concentration/size of carboxylated was determined as shown in Fig. S1.

### Animals

All the animal experiments strictly adhered to the standards of the institutional guidelines for ethics in animal experimentation (Rule number 86/609/EEC-24/11/86). All the experimental procedures were approved by the Animal Ethics Committee of Shanghai Jiao Tong University. In our experiments, a total of 12 healthy and sexually mature Duroc boars (2–3 years old) with proven fertility were selected from Shanghai Sunsing Livestock Co., Ltd. (Shanghai, China). All the chemical products were obtained from Sigma-Aldrich (St. Louis, MO, USA) unless otherwise mentioned.

### Semen Collection and Processing

After collection from twelve boars, a computer-assisted semen analysis (CASA) system (Hamilton Thorne Research, Beverly, MA, USA) was used to evaluate sperm motility. Only ejaculates with motility > 70% were included in this study. Each ejaculate was diluted in basal medium containing the following compounds: 27.5 mg mL^−1^ d-fructose, 6.9 mg mL^−1^ trisodium citrate dihydrate, 2.35 mg mL^−1^ ethylenediaminetetraacetic disodium salt, 1.0 mg mL^−1^ sodium hydrogen carbonate, 2.9 mg mL^−1^ citric acid monohydrate, 5.65 mg mL^−1^ tris (hydroxymethyl) aminomethane, 2 mg mL^−1^ skim milk, and 0.2 mg mL^−1^ amikacin sulfate. Skim milk (Foodhold USA LLC, Landover, MD, USA) was prepared in advance by sonication on ice for 40 min using an ultrasonic cell crusher (Hielscher Ultrasonics Gmbh, UP50H, Germany) (amplitude: 80%, cycle: 0.5).

In our experiments, we designed four experimental groups. Group I: carboxylated C_60_ was added to a final concentration of 1, 2, 3, or 4 μg mL^−1^ in the basal medium. In this group, we analyzed sperm motility, acrosome integrity, mitochondrial membrane potential, antioxidation ability, ATP level, and protein phosphorylation. Group II: different concentrations of H_2_O_2_ (0, 10, 100, 200, or 300 μM) were added to the basal medium that contained 2 μg mL^−1^ C_60_. Group III: different concentrations of C_60_ (0, 1, 2, 3, or 4 μg mL^−1^) were added to the basal medium that contained 100 μM H_2_O_2_. Group IV: 100 μM H_2_O_2_ was added to the basal medium in the presence or absence of 1.0 mM dbcAMP, or 2 μg mL^−1^ carboxylated C_60_ was added to the basal medium in the presence or absence of 0.1 mM H-89. In groups II, III and IV, we assessed sperm protein phosphorylation. All the semen samples reached a final concentration of 1 × 10^8^ cells mL^−1^ and were transported to the laboratory at 37 °C within 20 min. Upon arrival at the laboratory, the semen samples were immediately stored at 4 °C in an incubator sterilized with 75% alcohol. After 2–3 h, the temperature of the semen samples slowly decreased to 4 °C. During the incubation process, the samples were shaken gently three times a day to prevent precipitation.

### Sperm Quality Assessment

#### Sperm Motility

On each experimental day (days 3, 5, 10, and 15), 500 μL of each sample was taken from each bottle and incubated in a water bath at 37 °C for 30 min. After incubation, 5 μL of every 500-μL semen sample was placed on pre-warmed disposable counting chamber slides (Leja, Nieuw Vennep, the Netherlands). The computer-assisted semen analysis (CASA) system was used to measure sperm kinetic parameters [30]. The analysis was performed with images from several fields containing at least 200 spermatozoa per sample, and the sample analysis was based on the examination of 25 consecutive digitalized images.

#### Acrosome Integrity

Acrosome integrity was assessed after staining the sperm with fluorescein isothiocyanate-conjugated peanut agglutinin (PNA-FITC) as a marker of acrosomal status and propidium iodide (PI) as an indicator of live or dead sperm. On each experimental day (days 0, 3, 5, 10, and 15), 30 μL of each sample was taken from each bottle and collected by centrifugation at 2500×*g* for 5 min. After the removal of the supernatant, the pellets were suspended in 500 μL of phosphate-buffered solution (PBS) (8 g NaCl, 0.2 g KH_2_PO_4_, 1.15 g Na_2_HPO_4_, and 0.2 g KCl, diluted with double-distilled H_2_O to 1 L). The pellets were resuspended and incubated in the dark at 37 °C for 20 min with 2 μl PNA-FITC (100 μg mL^−1^) and 2 μL PI (1 mg mL^−1^). After incubation, each sample was centrifuged (2000×*g*, 5 min) and washed once with PBS. Then, 500 μL of PBS was added to resuspend each sample before flow cytometry analysis (Beckman Coulter Ltd., Brea, CA, USA) [31]. The results were expressed as the percentage of viable spermatozoa with an intact acrosome (FITC negative/PI negative).

#### Mitochondrial Membrane Potential

The specific probe 5,5′,6,6′-tetrachloro-1,1′,3,3′-tetraethylbenzimidazolyl carbocyanine iodide (JC-1) (Beyotime Institute of Biotechnology, Shanghai, China) was used to test the variation in mitochondrial membrane potential (Δ*ψ*_m_). The cells were stained with 0.5 mL JC-1 working solution (50 µL JC-1 (200 ×), 8 mL DDH_2_O, 2 mL JC-1 staining solution (50 ×) and incubated in the dark at 37 °C for 30 min. Following incubation, the samples were washed three times with 1 × assay buffer (2 mL JC-1 staining solution (50 ×, 8 mL double-distilled H_2_O), and the observed fluorescent signals were recorded using flow cytometry [32]. The FL-1 channel was used to detect JC-1 monomers and shows the number of sperm cells at a high membrane potential. The FL-2 channel was used to detect JC-1 aggregates and show the number of sperm cells at a low membrane potential. FL2/FL1 served as the value of Δ*ψ*_m_ [33].

#### Total ROS (tROS) Assay

The tROS levels of each semen sample were evaluated using the probe 2′, 7′-dichloride-hydrofluorescein diacetate (DCFH-DA, Beyotime Institute of Biotechnology, Nanjing, China). Intracellular DCFH-DA was oxidized by ROS to produce dichlorofluorescein with strong fluorescence. On each experimental day (days 0, 3, 5, 10, and 15), the semen samples were washed three times with PBS, resuspended, and incubated with 10 μM DCFH-DA at 37 °C in the dark for 30 min. The fluorescence intensity could be conveniently monitored using a fluorescence spectrophotometer at *E*_x_/*E*_m_ = 485/535 nm [34].

#### Total Antioxidative Capacity (T-AOC) Activity Assay

T-AOC activity was quantified using a T-AOC assay kit (Nanjing Jiancheng Bioengineering Institute, Jiangsu, China). The sperm samples were washed three times and resuspended in DDH_2_O. The suspension was lysed ultrasonically (20 kHz, 750 W, operating at 40% for 3 s, off for 5 s, 5 cycles) on ice and centrifuged (12,000×*g*, 10 min) at 4 °C. Supernatants were collected and mixed with the reaction buffer. Finally, a spectrophotometer was used to measure the T-AOC activity at 520 nm [35]. The T-AOC activity of each semen sample was converted into units per mL of total protein in spermatozoa and expressed as U mL^−1^.

#### Malondialdehyde (MDA) Content Assay

We used an MDA test kit (Nanjing Jiancheng Bioengineering Institute, Jiangsu, China) to measure the MDA content according to the manufacturer’s protocol. Briefly, on each experimental day (days 0, 3, 5, 10, and 15), the extracts of sperm were prepared by sonication (20 kHz, 750 W, operating at 40%, on 3 s, off 5 s, 5 cycles) in ice-cold buffer [50 mM Tris–HCl (pH 7.5), 5 mM EDTA, and 1 mM DTT]. The lysed cells were centrifuged (12,000×*g*, 10 min) to remove debris. Finally, the change in absorbance at 532 nm was measured using a spectrophotometer [35]. The sample MDA levels were recorded as nmol mL^−1^.

### Measurement of ATP Content and Safety Evaluation of Carboxylated C_60_

The ATP concentrations were measured using a bioluminescence ATP assay kit. On each experimental day (days 0, 3, 5, 10, and 15), 1 mL of each sample was collected from each bottle and washed twice with PBS. The lysis buffer was then added to the semen sample to extract the intracellular ATP. The luciferase reagent was mixed with the ATP extracts and serial dilutions of the ATP standard solution, and a luminometer (Thermo Fisher Evolution 300) was used to capture the fluorescent signals. The fluorescence values were compared with those of the standard ATP dilutions [36]. The safety of carboxylated C_60_ was evaluated by the conventional artificial insemination and reproductive index of sows.

### SDS-PAGE and Immunoblotting

One milliliter of each sample was collected from each bottle and centrifuged at 12,500×*g* for 6 min for four times; the samples were resuspended in 100 μL 5 × sample buffer and boiled for 4 min. The boiled samples were centrifuged (13,500×*g*, 10 min), and the supernatant was transferred to a new centrifuge tube. Aliquots of 10% β-mercaptoethanol were added to the samples, and the resulting sample mixture was boiled again for 3 min. The extracted sperm proteins were quantified by spectrophotometry (NanoDrop 2000, Thermo Scientific, USA) [27].

The extracted sperm proteins were resolved by SDS-PAGE. The resolved proteins were transferred onto PVDF membranes (Merck Millipore, Germany) at 90 V for 2 h on ice. The membranes were blocked with 1% BSA or 5% skim milk in T-TBS at room temperature for 1 h. T-TBS (1 ×) was then used to wash the membranes three times. After washing, the membranes were immunoblotted with anti-P-PKA antibody (Cell Signaling Technology, Cat# 9624, clone 100G7E) or anti-phosphotyrosine antibody (Millipore, Cat# 05-321, clone 4G10) followed by overnight incubation at 4 °C. The membranes were washed twice with T-TBS (1 ×) and then incubated with the corresponding secondary antibodies for 1 h at room temperature. An enhanced chemiluminescence ECL-plus kit (GE Healthcare Worldwide, USA) was used to develop the signals, which were detected using a ChemiScope 3300 mini-integrated chemiluminescence imaging system (CLINX, China). For all the experiments, the molecular weights of the sperm proteins are indicated in kDa [27].

### Immunofluorescence

The semen samples were collected, centrifuged at 800×*g* for 10 min, and resuspended in 500 μL of PBS. A total of 20 μL of each semen sample was placed on slides, smeared, and air-dried for 1 h. Then, 3.7% formaldehyde in PBS was used to fix the sperm for 20 min. After washing three times with PBS, the sperm were permeabilized with 0.5% Triton X-100 in PBS for 10 min. The semen samples were then washed five times with PBS and blocked with 1% BSA in PBS for 2 h at room temperature. After the slides were washed three times, the sperm were incubated with the anti-P-PKA antibody or anti-phosphotyrosine antibody at 4 °C overnight. After incubation, the sperm were washed with PBS and incubated with Alexa 555-conjugated anti-rabbit antibody (Molecular Probes, Cat# A-21428) or Alexa 488-conjugated anti-mouse antibody (Molecular Probes, Cat# A-11001) in 1% BSA in PBS for 2 h at room temperature; these solutions contained Alexa 488-conjugated PNA (Molecular Probes, Cat# L-21409) (1:100) for staining acrosomes and DAPI (1:100) for detection of the sperm nuclei. After 2 h of incubation, the sperm were washed four times and mounted on slides with an antifade solution. Finally, a confocal fluorescence microscope (Leica, TCS, SP5) equipped with a 400 × objective was used to examine the sperm samples [27].

### Statistical Analysis

All data are indicated as the mean ± standard error of the mean (SEM). The variances were first analyzed using a homogeneity test. If the data met the assumption of homoscedasticity, the significance of differences in means was determined by one-way analysis of variance (ANOVA) followed by an LSD *t* test for multigroup comparisons. Otherwise, significance was determined by the Tamhane’s T2 test. All the statistical analyses were performed using SPSS 17.0 software. A *P* value < 0.05 was considered statistically significant.

## Results and Discussion

### The Effect of Carboxylated C_60_ on Boar Sperm Quality

**Fig. 1:**
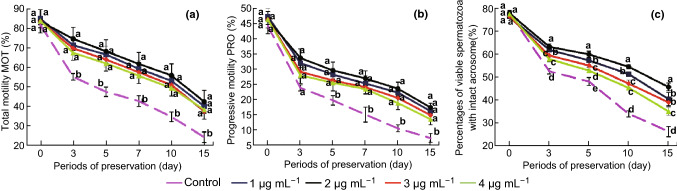
Effects of carboxylated C_60_ on motility, acrosome integrity, and mitochondrial membrane potential of boar semen preserved at 4 °C for half a month. **a** Sperm motility parameters (MOT), **b** progressive motility parameters (PRO), **c** acrosome integrity. Each experiment was performed at least six times and subjected to statistical analysis. Different lowercase letters within the same day demonstrate significant differences (*P* < 0.05), whereas the same lowercase letters denote insignificant differences

The effects of different concentrations of carboxylated C_60_ supplementation on boar sperm motility during liquid storage at 4 °C are shown in Fig. 1a. The results indicated that supplementation with 1, 2, 3, or 4 μg mL^−1^ carboxylated C_60_ could significantly improve the sperm motility parameters when compared with those of the control. Moreover, no significant difference was observed among the different treatment groups (*P *> 0.05). Total motility (MOT) and progressive motility (PRO) showed higher levels throughout the entire storage time in the treatment groups than in the control group (*P *< 0.05) (Fig. 1a, b). Moreover, since an intact acrosome in viable sperm is crucial to perform the acrosome reaction, acrosome integrity is another key performance indicator to assess sperm quality. As shown in Fig. 1c, the percentage of viable spermatozoa with an intact acrosome significantly increased in the carboxylated C_60_-treated group compared with that in the untreated group (*P *< 0.05). Additionally, throughout the experimental periods, with regard to acrosome integrity, the 2 μg/mL-carboxylated C_60_-treated group performed better than the 3 μg/mL- or 4 μg/mL-carboxylated C_60_-treated groups. After 15 days of preservation, the percentage of acrosome integrity in the 2 μg/mL-carboxylated C_60_-treated group was higher than in the 1, 3, and 4 μg/mL-carboxylated C_60_-treated groups (*P *< 0.05). Taken together, supplementation with 2 μg mL^−1^ carboxylated C_60_ had the strongest protective effect on the sperm acrosome.

### The Effect of carboxylated C_60_ on Boar Sperm Antioxidation Ability

**Fig. 2:**
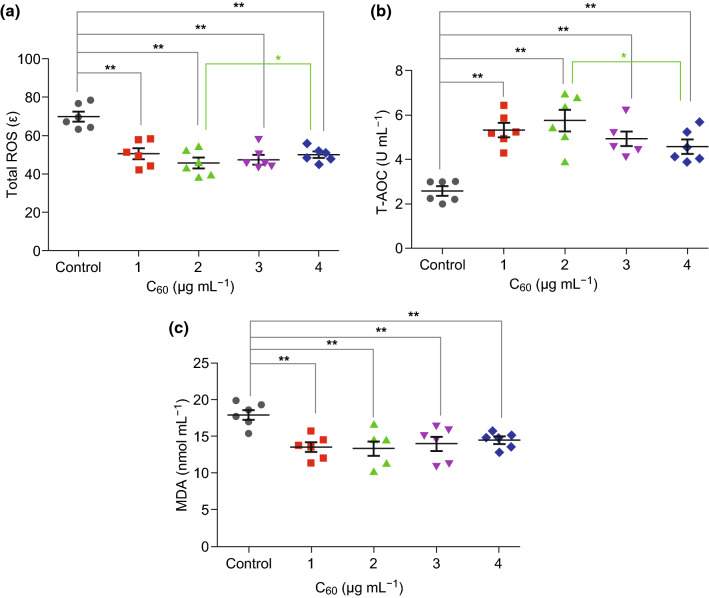
Dot plots of the tROS level, T-AOC activity, and MDA content, and ATP levels in the control and treatment groups after 15 days of treatment. **a** tROS level, **b** T-AOC activity, **c** MDA content (*n* = 6, *P < 0.05, **P < 0.01)

Previous studies have demonstrated that sperm motility, acrosome integrity, and lipid peroxidation are more sensitive indicators of oxidative stress [37, 38], and ROS levels can be managed by the addition of ROS-scavenging antioxidants [39]. Therefore, the protection of spermatozoa against oxidative stress by antioxidant supplementation is currently pursued [40]. To verify the potential protective effects of carboxylated C_60_ against oxidative damage, we analyzed the effects of carboxylated C_60_ in the oxidative and antioxidative states by measuring the tROS level, T-AOC activity, and MDA content. As shown in Fig. 2, when compared with the control group, T-AOC activity in the carboxylated C_60_-treated groups was significantly increased after 15 days of preservation (*P *< 0.01), while the MDA content and tROS level were dramatically decreased (*P *< 0.01). Meanwhile, the 2 μg/mL-carboxylated C_60_-treated group had lower total ROS and higher T-AOC than the 4 μg/mL-carboxylated C_60_-treated group (*P* < 0.05). Given the above findings, it is reasonable to surmise that carboxylated C_60_ treatment could maintain the antioxidative capacity of boar sperm and suppress the accumulation of ROS and MDA.

In the last decade, studies have indicated that carboxylated C_60_, due to the physicochemical properties of its spherical cage-like molecule built exclusively from carbon atoms, is able to scavenge reactive oxygen species. Carboxylated C_60_ can efficiently protect the central nervous system from oxidative stress-induced damage [17]. Likewise, it can protect DNA from ionizing radiation-induced oxidative damage in vitro [16]. In particular, it is worth mentioning that carboxylated C_60_ has been demonstrated to reduce diabetes-induced oxidative stress and associated complications and testicular dysfunction [15]. Consistent with these results, we also determined that carboxylated C_60_ could protect boar sperm against oxidative damage during liquid storage at 4 °C. This finding could explain why carboxylated C_60_ exhibited antioxidant capacity at low concentrations and small doses, further providing a theoretical basis for the present study.

Hence, the obtained data sufficiently expand our knowledge concerning the potential involvement of fullerene nanostructures in processes that occur in sperm at 4 °C in vitro. Indeed, based on their antioxidant properties, fullerenes have been shown to have protective effects in various cell types [41–43] and within multifarious disease contexts [44–47]. It is worth mentioning that carboxylated C_60_ has recently been demonstrated to be able to inactivate free radicals (ROS) with values that substantially exceed the theoretically expected level. Fullerene C_60_ has been observed to exert pronounced testicular-protective efficacy in preventing oxidative stress induced by doxorubicin [48]. This result is consistent with previous studies that have reported that carboxylated C_60_ prevents Fe^2+^/ascorbate-induced oxidative stress in goat epididymal spermatozoa in vitro [49]. Although much attention has been given to the effects of carboxylated C_60_ on the reproductive health in experimental animals, it has not been investigated whether carboxylated C_60_ has a protective effect on sperm. Therefore, the present study remains the first to reveal carboxylated C_60_ as a protective antioxidant against ROS toxicity in boar sperm preserved at low temperatures.

### The Effect of Carboxylated C_60_ on Boar Sperm ATP Levels

**Fig. 3:**
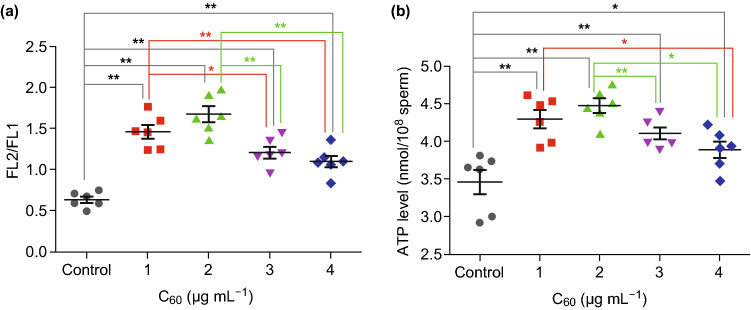
Dot plots of the mitochondrial membrane potential and ATP in the control and treatment groups after 15 days of treatment. **a** Mitochondrial membrane potential, **b** ATP level (n = 6, *P < 0.05, **P < 0.01)

Mitochondrial membrane potential (Δ*ψ*_m_) is used to characterize the structural and functional integrity of mitochondria [50]. As indicated in Fig. 3a, after 15 days of preservation, the Δ*ψ*_m_ values of the treatment groups were higher than that of the control group (*P *< 0.01). Moreover, with the increasing in vitro storage time, the groups treated with 1 and 2 μg mL^−1^ carboxylated C_60_ showed higher Δ*ψ*_m_ values than those of the 3 and 4 μg/mL-carboxylated C_60_ treatments (*P *< 0.05 and *P *< 0.01, respectively); no significant difference was observed between the two groups.

It is generally accepted that intracellular ATP, which is an indicator of sperm motility, is required to maintain sperm flagellar movement and signal transduction [36, 51]. Therefore, the ATP levels of boar sperm treated with different concentrations of carboxylated C_60_ were investigated. The results indicated that the untreated group had the lowest ATP level among all the groups with increasing in vitro storage time (*P *< 0.01) (Fig. 3b). This result was in agreement with the sperm motility. Moreover, the ATP level was higher in both 1 and 2 μg/mL-treated groups than that in untreated group, and no difference was observed between these two treatment groups. Furthermore, the safety of carboxylated C_60_ as an alternative antioxidant was comprehensively evaluated using artificial insemination. Regardless of the mean litter size or the mean number of live offspring, the carboxylated C_60_ treatment group showed an increasing trend, although there was no significant difference with respect to the control group (Fig. S2).

### The Effect of Carboxylated C_60_ on Protein Phosphorylation

**Fig. 4:**
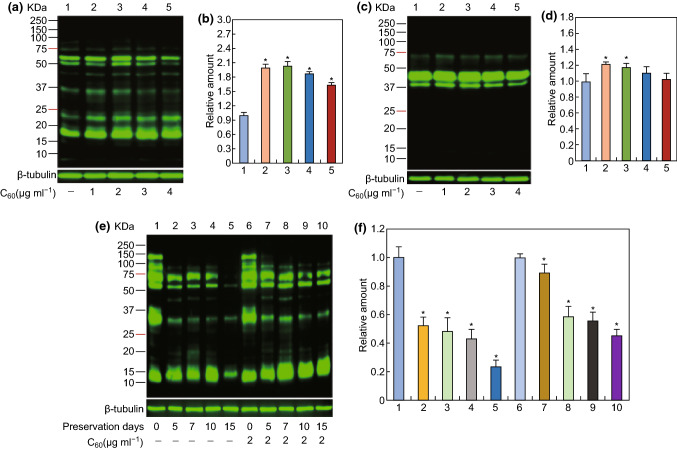
Western blot analysis of P-PKAs and PTP under different concentrations of carboxylated C_60_. Western blot analysis was performed using **a**, **b** an anti-phospho-PKA substrate antibody, **c**, **d** an anti-phosphotyrosine antibody, **e**, **f** an anti-phospho-PKA substrate antibody. The bands used for histogram quantification are labeled #. β-Tubulin was used as an internal control. The experiment was performed at least three times; the presented image is representative and repeatable. (n = 3, *P < 0.05)

**Fig. 5:**
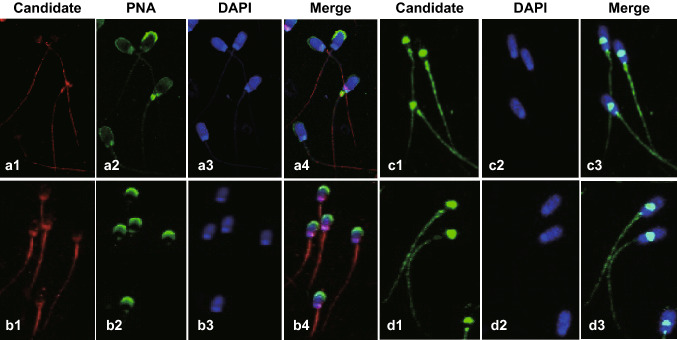
Immunolocalization of phosphorylated PKA substrates (a1–4 are the control; b1–4 are in the presence of 2 μg mL^−1^ carboxylated C_60_) and tyrosine-phosphorylated (c1–3 are the control; d1–3 are in the presence of 2 μg mL^−1^ carboxylated C_60_) proteins in boar sperm. Sperm were incubated in the basal medium in the presence or absence of 2 μg mL^−1^ carboxylated C_60_ with primary anti-P-PKAs and anti-phosphotyrosine antibody. PNA (peanut agglutinin) was applied to stain the acrosome and DAPI to stain the nuclei of sperm. The merged images are also shown. The experiment was performed at least three times, and the image presented is representative and repeatable. Sperm cells were visualized using a confocal laser scanning microscope (× 400)

In addition to the supply of ATP, protein phosphorylation is another factor affecting sperm motility [52]. It has been well documented that phosphorylation of PKA substrates (P-PKAs) and protein tyrosine phosphorylation (PTP) are closely related to the cAMP-PKA signaling pathway, which is responsible for the regulation of sperm motility [53]. Although many previous studies have examined protein phosphorylation in sperm, whether carboxylated C_60_ impacts sperm protein phosphorylation modifications is still not completely understood. Thus, we measured protein phosphorylation in boar sperm and found that it was affected by a series of carboxylated C_60_ concentrations. The results showed that the level of P-PKAs was noticeably higher in the treatment groups than that in the control group (*P* < 0.05) (Fig. 4a, b). In addition, compared with the other treatment groups, supplementation with 2 μg mL^−1^ carboxylated C_60_ had the most remarkable impact on P-PKAs (*P* < 0.05) (Fig. 4a, b). We also measured the effect of carboxylated C_60_ on PTP and obtained approximately similar findings (Fig. 4c, d). The changes in PTP in the 1, 2, and 3 μg/mL-carboxylated C_60_ treatment groups were, as expected, greatly enhanced compared with that in the control group and the 4 μg/mL-carboxylated C_60_ treatment group (*P* < 0.05). Moreover, the most significant increase in the level of PTP was observed in sperm incubated with 1 or 2 μg mL^−1^ carboxylated C_60_ (*P* < 0.05) (Fig. 4c, d). Consistently, these results demonstrated that the optimal dose of carboxylated C_60_ was 2 μg mL^−1^. Additionally, immunolocalization of P-PKAs and tyrosine-phosphorylated proteins in boar sperm also demonstrated the promotion of protein phosphorylation by carboxylated C_60_, in accordance with the western blot results (Fig. 5). In the present study, our results showed that the phosphorylation level decreased alongside decreased spermatozoa motility, indicating that the phosphorylation levels in sperm protein positively correlated with the motility parameter [54].

In addition, to further explore the molecular mechanism underlying the protective roles of carboxylated C_60_ from the perspective of protein phosphorylation, we analyzed the trend in P-PKAs between the control and 2 μg/mL-carboxylated C_60_ treatment groups after specific periods of preservation. As described in Fig. 4e, f, the results revealed a decrease in the level of P-PKAs in both groups as the incubation progressed. Interestingly, a higher level of P-PKAs was detected in the 2 μg/mL-carboxylated C_60_ treatment group than in the control group after the same period of preservation. Therefore, our results indicate that carboxylated C_60_ affected protein phosphorylation, at least in part, by inhibiting protein dephosphorylation in boar sperm.

### Carboxylated C_60_-Induced Promotion of Protein Phosphorylation and Antioxidative Capability

**Fig. 6:**
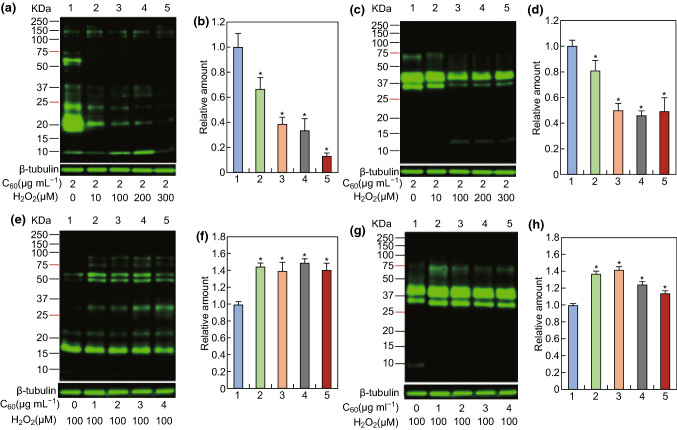
C_60_-induced P-PKAs and PTP were affected by a series of H_2_O_2_ concentrations. **a**, **b** Western blot analysis was performed using an anti-phospho-PKA substrate antibody. **c**, **d** Western blot analysis was performed using an anti-phosphotyrosine antibody. Spermatozoa were incubated in basal medium containing 2 μg mL^−1^ carboxylated C_60_ and a series of H_2_O_2_ concentrations (0, 10, 100, 200, and 300 μM). β-Tubulin was used as an internal control. **e**, **f** Western blot analysis was performed using anti-phospho-PKA substrate antibody. **g**, **h** Western blot analysis was performed using anti-phosphotyrosine antibody. The experiment was performed at least three times, and the image presented is representative and repeatable. (n = 3, *P < 0.05)

It is noteworthy that sperm proteins are targets of redox-dependent modifications, which seem to play a much greater role, leading, depending on the levels of ROS, either to the activation/inactivation of signaling pathways that are important for sperm physiology or to the oxidative damage and impairment of vital functions [55]. Moreover, it has been previously shown that suitable ROS levels are essential for the level of protein phosphorylation [56]. Low levels of ROS induce protein phosphorylation [57]. In contrast, high levels of ROS inhibit the synthesis of sAC, reduce intracellular cAMP levels, and inhibit protein phosphorylation [58]. To examine the effects of intracellular ROS on protein phosphorylation in boar sperm, we used the oxidant H_2_O_2_ to simulate mitochondrial ROS. The results showed that as the H_2_O_2_ concentration increased, the level of P-PKAs decreased in a dose-dependent manner (Fig. 6a, b). When the H_2_O_2_ concentration was increased to 300 μM, the level of P-PKAs became very low. In contrast, the level of tyrosine phosphorylation decreased as the H_2_O_2_ concentration increased up to 100 μM and then remained stable at H_2_O_2_ concentrations > 100 μM (Fig. 6c, d). To further test whether carboxylated C_60_ could eliminate excess mitochondrial ROS to regulate protein phosphorylation, we performed another experiment using H_2_O_2_. As described in Fig. 6e–h, when 1, 2, 3, or 4 μg mL^−1^ carboxylated C_60_ was added to the basal medium in the presence of 100 μM H_2_O_2_, the levels of P-PKAs and PTP were all noticeably higher than in the control. Typically, 2 μg mL^−1^ might be the optimum concentration of carboxylated C_60_ to restore protein phosphorylation levels. These data suggested that the antioxidative ability of carboxylated C_60_ prevented H_2_O_2_ from inhibiting protein phosphorylation.

**Fig. 7:**
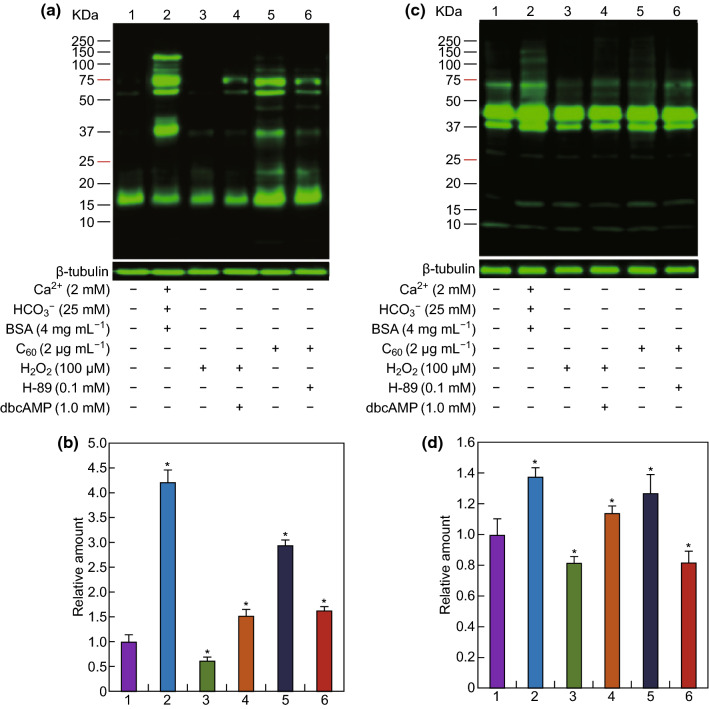
Effects of the cAMP-PKA pathway-related regulatory factors on P-PKAs and PTP with different concentrations of carboxylated C_60_. **a** Western blot was performed using an anti-phospho-PKA substrate antibody. **b** Densitometric analysis of the western blot image shown in **a**. **c** Western blot was performed using an anti-phosphotyrosine antibody. **d** Densitometric analysis of the western blot image shown in **c**. Spermatozoa were incubated in basal medium supplemented with 100 μM H_2_O_2_ in the presence or absence of 1.0 mM dbcAMP, or 2 μg mL^−1^ carboxylated C_60_ in the presence or absence of 0.1 mM H-89. β-Tubulin was used as an internal control. The experiment was performed at least three times, and the image presented is representative and repeatable. (n = 3, *P *< 0.05)

To further investigate whether the promotion of protein phosphorylation by different concentrations of carboxylated C_60_ occurred via the cAMP-PKA signaling pathway, the sperm were cultured with the cAMP analog dibutyryl-cAMP (dbcAMP) in the presence or absence of 100 μM H_2_O_2_, or the PKA inhibitor H-89 in the presence or absence of 2 μg mL^−1^ carboxylated C_60_. The results showed that inhibition of protein phosphorylation by 100 μM H_2_O_2_ could be reversed by the addition of 1.0 mM dbcAMP (Fig. 7). In addition, protein phosphorylation was decreased by supplementation with H-89 compared with that in the carboxylated C_60_-treated group (Fig. 7). The results indicated that carboxylated C_60_ affected protein phosphorylation, at least in part through the cAMP-PKA pathway in boar sperm.

To our knowledge, this is the first exploration of the molecular mechanism underlying the protective action of carboxylated C_60_ against ROS toxicity, which results from the oxidative stress and energy deficiency, by inhibiting the protein dephosphorylation caused by ROS via the cAMP-PKA signaling pathway. The data suggest that carboxylated C_60_ nanoparticles may eliminate ROS from sperm cells, resulting in reduced levels of intracellular ROS, thus preventing mitochondrial damage and improving the motility parameters. Importantly, these discoveries contribute to a more comprehensive view of the molecular mechanisms underlying the protective effects of exogenous antioxidants on sperm and indicate the practical feasibility of using carboxylated C_60_ as a boar semen extender supplement for assisted reproductive technology.

### The Interactions between Sperm and Carboxylated C_60_

**Fig. 8:**
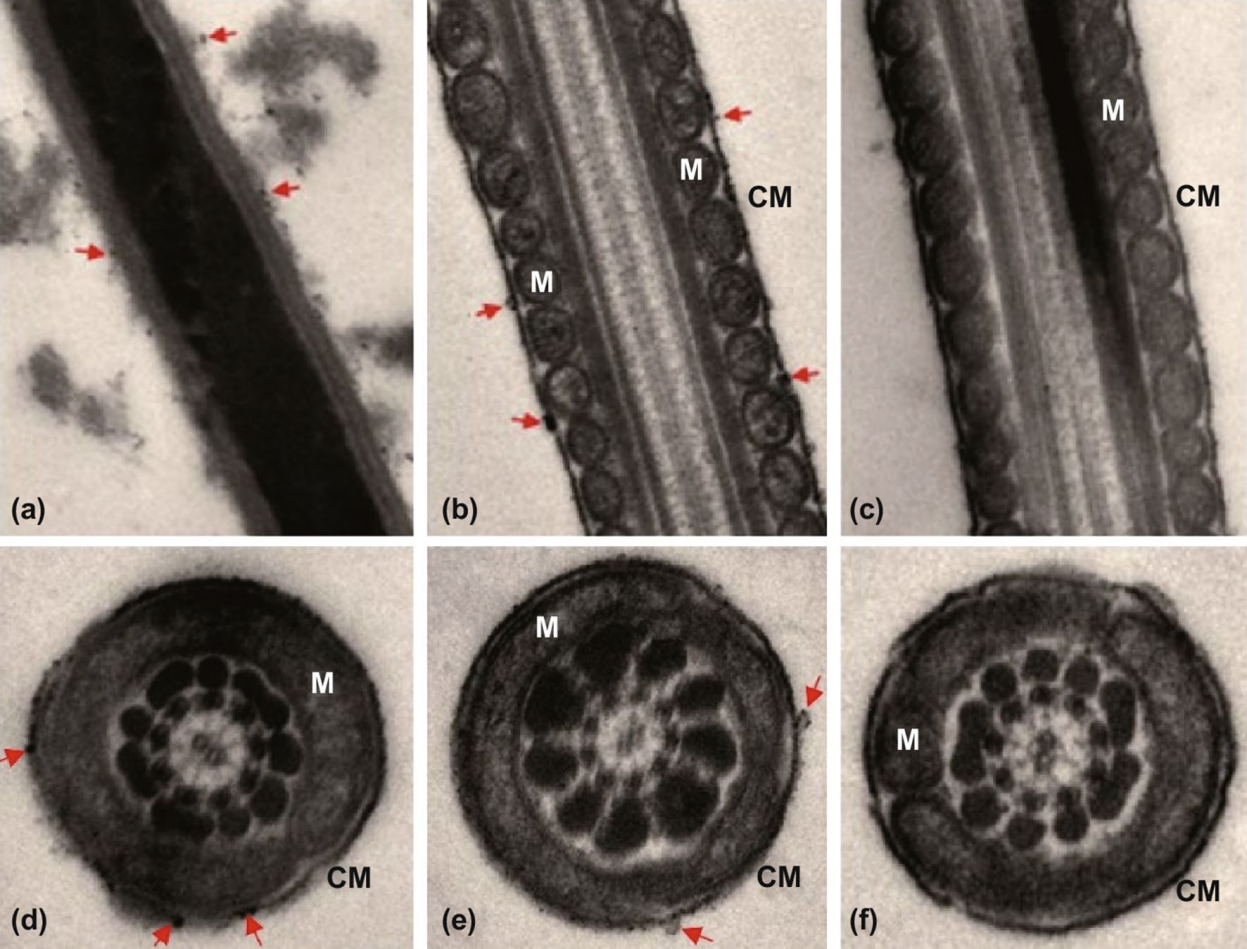
TEM images of spermatozoa samples treated with carboxylated C_60_ at 4 °C. The spermatozoa were collected after 15 days of treatment. TEM images showing **a** the sperm head interacting with carboxylated C_60_ (2 μg mL^−1^), **b** the sperm midpiece interacting with carboxylated C_60_ (2 μg mL^−1^), **c** the sperm midpiece without carboxylated C_60_. **d**, **e** Cross-sectional TEM images showing the sperm midpiece interacting with carboxylated C_60_ (2 μg mL^−1^). **f** Cross-sectional TEM image showing the sperm midpiece without carboxylated C_60_. CM: cell membrane; M: mitochondria

To observe how carboxylated C_60_ interacted with sperm cells, samples were examined by TEM (Fig. 8). Carboxylated C_60_ attached to sperm primarily as single nanoparticles or aggregates on the sperm plasma membrane (Fig. 8). We re-examined the particle diameter in the TEM images and found that primary particle diameters were similar to the size of carboxylated C_60_ (75.5 ± 7.2 nm) by NanoSight analysis (Fig. S1). No nanoparticles were observed to penetrate morphologically intact sperm in any case.

**Fig. 9:**
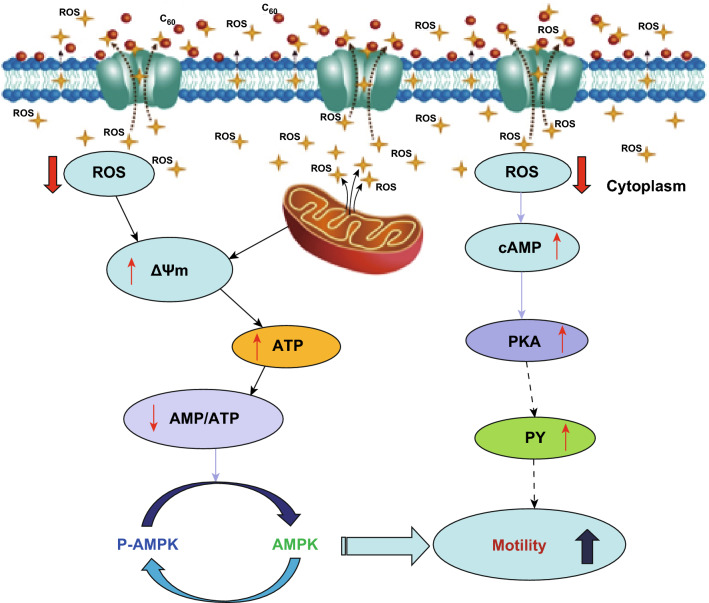
Putative mechanisms by which carboxylated C_60_ protects boar sperm from ROS-induced functional damage at 4 °C. Carboxylated C_60_ may bind to ROS outside and reduce ROS levels inside sperm. Decreasing intracellular ROS levels may enhance the mitochondrial membrane potential and cAMP levels and also reduce cellular protein dephosphorylation and enhance cellular ATP levels, subsequently increasing the motility of spermatozoa at 4 °C. Δ*ψ*_*m*_: mitochondrial membrane potential; PKA: protein kinase A; PY: tyrosine phosphorylation

Interestingly, the present data suggest that the protective effects of carboxylated C_60_ on boar spermatozoa can be attributed to its antioxidant activity, resulting in an advanced fertility capacity of sperm cells during preservation at 4 °C. Numerous experimental studies have been conducted to evaluate the application of various antioxidants to protect spermatozoa against the oxidative damage caused at low temperatures [27, 37]. Our findings demonstrated that carboxylated C_60_ protected mitochondria by scavenging ROS and increasing antioxidant ability, which might be related to the unique pseudo-aromatic structure of carboxylated C_60_, resulting in delocalization of π-electrons over its carbon core and readily reactivity with oxygen free radicals. Over the last decade, the probable function mechanism of carboxylated C_60_ has been extensively studied. Carboxylated C_60_ is highly hydrophilic and a highly stable donor–acceptor complex of C_60_ with water molecules—C_60_@{H_2_O}_*n*_, *n* = 22–24 [59]. The currently recognized carboxylated C_60_ antioxidant mechanism has the following aspects: carboxylated C_60_ as a novel “structural” antioxidant that is characterized as a “radical sponge” [60], the enzyme catalysis mechanism [61], the stabilizing effects on alkaline phosphatase and peroxidase in vitro [13], and the binding of free radicals and removal of hydroxyl radicals [16]. However, TEM analysis revealed a modification-dependent attachment of carboxylated C_60_ to the cell membrane of spermatozoa (Fig. 8), but provided no evidence of the entrance of carboxylated C_60_ nanoparticles into sperm cells. Therefore, the results obtained in the present study suggest an alternative mechanism of the antioxidative activity of carboxylated C_60_ that results in ROS spillover in sperm cells, which could explain the decrease in sperm ROS levels (Fig. 9). We hypothesized that the decrease in intracellular ROS levels in spermatozoa caused by carboxylated C_60_ is related to the surface electron affinity. Additionally, antioxidant ability related to an extended electron-conjugation system only determined the high reactivity toward ROS.

## Conclusions

To our knowledge, we report, for the first time, that carboxylated C_60_ can be used as a safe antioxidant agent to serve as semen extender supplement and that such a practice can improve the survival and characteristics of boar sperm during liquid storage. Our results suggested that carboxylated C_60_ could effectively protect boar sperm against oxidative injury. This approach can represent a good alternative to the preservation methods used for boar semen and anti-sterility drugs used for other reproductive diseases. Our results provide reliable theoretical support for the future application of carboxylated C_60_ in breeding livestock boar. Furthermore, the results present the first substantial evidence that carboxylated C_60_ administration significantly reduces the oxidative stress on boar spermatozoa stored at 4 °C and the associated complications, similar to a semen extender supplement.

## Electronic Supplementary Material

Below is the link to the electronic supplementary material.

Supplementary material 1 (PDF 200 kb)
