# Supplementary material for: C60 Fullerenes Suppress Reactive Oxygen Species Toxicity Damage in Boar Sperm
Source: Nanomicro Lett. 2019 Nov 27;11:104. doi: 10.1007/s40820-019-0334-5 (PMC7770955; doi:10.1007/s40820-019-0334-5)
Supplement: Supplementary file 1 — Supplementary material 1 (PDF 200 kb) [file 40820_2019_334_MOESM1_ESM.pdf]

Supporting Information for

C_{60} Fullerenes Suppresses Reactive Oxygen Species Toxicity Damage in Boar Sperm

Xinhong Li[1,*], Lirui Wang[1,2], Jieli Fu[1], Linqing Zhen[1], Yuhua Li[1], Yaozhong Zhang[4], Yafei Zhang[3,*]

[1]Shanghai Key Laboratory of Veterinary Biotechnology, School of Agriculture and Biology, Shanghai Jiao Tong University, Shanghai 200240, People's Republic of China

[2]Institute of Nano Biomedicine and Engineering, Shanghai Engineering Research Centre for Intelligent Diagnosis and Treatment Instrument, Department of Instrument Science and Engineering, School of Electronic Information and Electrical Engineering, Shanghai Jiao Tong University, Shanghai 200240, People's Republic of China

[3]Key Laboratory of Thin Film and Microfabrication (Ministry of Education), Department of Micro/Nano Electronics, School of Electronics, Information and Electrical Engineering, Shanghai Jiao Tong University, Shanghai 200240, People's Republic of China

[4]Department of Electrical and Computer Engineering, Michigan State University, East Lansing, USA

*Corresponding authors. E-mail: lixinhong7172@sjtu.edu.cn (Xinhong Li); yfzhang@sjtu.edu.cn (Yafei Zhang)

Supplementary Figures

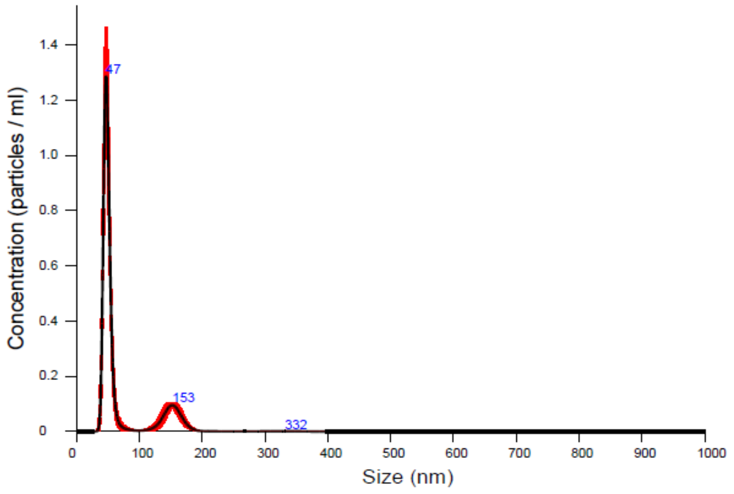

Fig. S1 The averaged FTLA concentration/size for carboxylated C_{60}

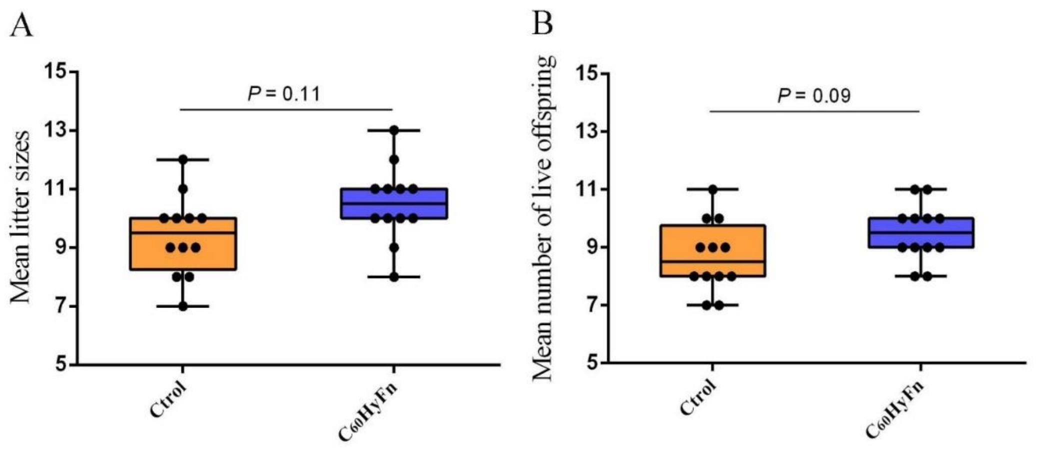

Fig. S2 The reproductive indexes of sows using the semen diluent supplement with carboxylated C_{60} were compared
